# The aggregate effects of government income transfers shocks: EU evidence

**DOI:** 10.1007/s13209-022-00271-x

**Published:** 2023-01-07

**Authors:** Susana Párraga Rodríguez

**Affiliations:** Banco de España, Alcalá 48, 28014 Madrid, Spain

**Keywords:** Transfer payments, Public pensions, Fiscal multiplier, European Union, E2, E62, H55, I38

## Abstract

This paper estimates the aggregate effects of government income transfers shocks for a sample of EU countries. I construct a new measure of transfers shocks based on a dataset by public finance experts of the European System of Central Banks (ESCB). The identification strategy consists of a narrative analysis of policy actions in old age pensions reported in the ESCB dataset. I find that increases in old age pensions have a positive impact on aggregate expenditure components and employment consistent with a multiplier effect between 0 and 1.

## Introduction

Governments around the world responded swiftly to the economic crisis caused by the covid-19 pandemic. Among the budgetary measures adopted to mitigate the adverse effects on households highlighted cash payments and the reinforcement of government income transfers (see, for example, Cuadro-Sáez et al. 2020; Liu et al. 2021; Kubota et al. 2021). Sound economic policy calls for a quantitative assessment of the adopted measures and benchmarks such as fiscal multipliers or the marginal propensity to consume (MPC). However, the question of what are the aggregate effects of government income transfers shocks has received comparatively little attention in the literature; see, for example, Ramey (2019) literature review of the renaissance in fiscal research in the last decade. This paper contributes to the existing literature estimating the aggregate impact of government income transfers shocks using a panel dataset of 22 EU Member States over the sample period 2007–2015. Specifically, I estimate the multiplier effect and the responses of aggregate expenditure components and labour market indicators to changes in old age pensions.

Empirical evidence on the subject is scarce and has focused on the effects that changes in income have on private consumption expenditures. In the framework of the permanent income hypothesis, Poterba (1988) estimates that a $1 increase in transitory income due to the US tax rebates of 1975 raised spending of non-durables and services by about 12–24 cents. Wilcox (1989) finds that a predictable 10% increase in US social security benefits raises durable goods purchases by 3% in the same month. Romer and Romer (2016) construct a series of legislated increases in social security benefits in the USA from 1951 to 1991 and study the effect of innovations to their narrative variable on private consumption. They find that permanent benefit increases have a significant impact on consumption upon impact. This paper complements previous work in Párraga-Rodríguez (2018, 2022) along three dimensions. First, while my previous research focused on a single-country analysis for the USA or Spain, this paper uses a sample of EU countries. Second, this paper estimates the aggregate effects of transfers shocks on an extended set of outcome variables which includes output, aggregate private consumption, investment, and several labour market indicators. Finally, like Gechert et al. (2016), the principal contribution of this research is an estimate for the transfers output multiplier. Like Gil et al. (2019), I use a narrative approach to identify the effects of fiscal policy. Like Oh and Reis (2012) I look at a recent sample period before the pandemic. However, while they focus on the expansionary side of fiscal policy actions in the USA between 2007 and 2009, my economic unit of reference are European countries and the sample period includes both stimulus plans and fiscal consolidations.

Evidence at the household level is much more prolific and indicates a positive response of individual spending to increases in government income transfers. Jappelli and Pistaferri (2010) offer a good literature review on the subject. Relevant studies include, for example, a pioneering quasi-experimental approach by Bodkin (1959). He looks at the consumption response of WW-II veterans after the receipt of unexpected transfer payments in 1950, and finds a marginal propensity to consume non-durables as high as 0.72. Hausman (2016) also looks at the consumption response of US veterans, but of WW-I, in a natural experiment setting. He finds that within six months of receiving a large bonus in June 1936, veterans spent between 0.65 and 0.75 cents out of every dollar received, and that they spent a large fraction of their bonus on cars, i.e. durable goods. Parker et al. (2013) exploit the randomisation in the assignation of Social Security numbers in the USA to estimate the effect of the tax rebates of 2008 on households spending. They find that on average households spent about 50–90% of their stimulus payments on durable goods (also mainly cars), and about 12–30% on non-durables consumption goods and services in the quarter of the tax rebate. The estimated spending responses are the largest for low-income, old age and borrowing constrained households.^1^ Stephens (2003) investigates the response of household consumption expenditures to the regular monthly arrival of social security checks in the USA. He finds an increase in the amount and probability of consuming strictly non-durables the immediate days after receiving the checks. The results are even more significant for those households for which social security transfers constitute their main source of income. Finally, in Párraga-Rodríguez (2022), I find that Spanish pensioners have a high marginal propensity to consume (MPC) due to unexpected permanent income increases, but less than the one-for-one responses predicted by the canonical permanent income hypothesis. Moreover, high spending responses by high-income and high-wealth pensioners, particularly on durables, discard liquidity constraints as a key source of MPC heterogeneity for pensioners.

Government income transfer shocks are constructed from a new and confidential dataset by public finance experts from the European System of Central Banks (ESCB). The dataset contains detailed information on public revenue and expenditure policies for several EU Member States. Within government income transfers, the data reports policy actions for old age pensions, unemployment benefits, and a residual category for other transfers. This paper though restricts the attention to old age pensions. This restriction is primarily due to a lack of observations of discretionary changes in unemployment benefits and, the difficult economic interpretation of estimates for other transfers due to the variety of benefits included in this category.^2^ The policy actions are reported with annual frequency following standardised questionnaires in the context of regular projection exercises; the data are harmonised across countries. The dataset defines a policy action as any change to legislation which determines benefit entitlements. Furthermore, fiscal actions are measured as the difference relative to a benchmark of neutral fiscal policy. The ESCB dataset compiles discretionary changes in fiscal policy.

The challenge for any study of the aggregate effects of fiscal shocks is the potential endogenous policy actions. Policymakers take policies for a variety of reasons. For example, during periods of high levels of inflation, governments may increase income transfer payments to guarantee the purchasing power of their beneficiaries. Another example is that in the event of a recession, extraordinary measures may be needed to help a growing number of unemployed. Then, on many occasions fiscal policy measures are responding to the current state of the economy. The key identifying assumption to produce unbiased estimates of the aggregate effect of transfers shocks is that discretionary changes in government income transfers are exogenous. The ESCB dataset records discretionary changes in transfers. A contribution of this paper is to reclassify these discretionary changes as either exogenous or not exogenous based on their motivation. To do so I use information contained in the descriptions accompanying all measures in the ESCB dataset. I complement this information with several other sources, including country-specific legislation and government reports, country reports by different international organisations, and the occasional newspaper.

I find a multiplier effect between 0 and 1. The estimated old age pensions output multiplier is 0.5 upon impact, with a maximum cumulative response close to the unity. Consistent with the existing literature (and household-level evidence) I also find a larger effect on durables consumption than non-durables or services. The response of investment is comparable to that of durables consumption. Moreover, increases in transfers have a positive though modest impact on employment. To gain insights into these results, estimates are also broken down by main motivation behind the policy actions and for three geographic regions, i.e. North, South and East Europe. Estimates by the motivation of the policies indicate similar positive aggregate effects. Regarding regional estimates, I find that the point estimates are only statistically significant for South Europe.

An estimate of the transfers multiplier effect is crucial for assessing the effectiveness of fiscal policy actions. A multiplier effect between 0 and 1 indicates limited effectiveness of fiscal actions involving government income transfers. However, this limited effectiveness has different implications for stimulus and austerity programmes. The results indicate that increases in old age pensions might be costly stimulus measures given their modest positive aggregate impact. On the other hand, desirable austerity programmes should include measures that effectively reduce the government deficit while having a contained negative effect on the economy.

The remainder of the paper is organised as follows. Section 2 describes the ESCB dataset and the construction of the new measure of transfers shocks. Section 3 gives details about the specification used for estimation. Section 4 explains the main results in terms of the multiplier effect and investigates the transmission mechanism of transfers shocks. Section 5 breaks down the estimates by motivation and economic region. Section 6 offers concluding remarks.

## A new measure of transfers shocks

A contribution of this paper is to construct a new measure of exogenous government income transfers shocks. I apply the narrative analysis pioneered by Romer and Romer (2010) to a new dataset compiled by public finance experts from the European System of Central Banks (ESCB).

### The ESCB dataset

The ESCB dataset compiles discretionary changes of fiscal policy. In the dataset, policy actions are any change to legislation which determines benefits entitlements. Moreover, policy actions are measured as the difference relative to a ‘neutral policy’ benchmark, i.e. policies follow the standard development. The benchmark for pensions adjustments is to report the measures in deviation from the price index of reference, once controlled for the evolution of beneficiaries. The benchmark for reforms is a hypothetical counterfactual of no change in the legislation. That is recorded as the difference in expenditure from what it would have been absent the change in the legislation. It is assumed the same dynamics as in the previous year.

As an example, Table 1 in appendix summarises the policy actions and methods reported in the ESCB dataset by Spain. The table includes the source, motivation, and description for all policy actions. Morris et al. (nd) provide more examples for other countries.

The ESCB dataset complements official/external sources with estimates by the public finance experts of the ESCB. The experts produced estimates whenever the impact of a measure was not available from official sources, the information provided by governments or other public agencies was insufficient or the actual macroeconomic and/or demographic situation deviated significantly from the assumptions made by the external source.

The EU Member States covered in this paper include Austria (AT), Belgium (BE), Bulgaria (BG), Cyprus (CY), The Czech Republic (CZ), Germany (DE), Spain (ES), Finland (FI), France (FR), Greece (GR), Hungary (HU), Ireland (IE), Italy (IT), Luxembourg (LU), Latvia (LV), Malta (MT), the Netherlands (NL), Poland (PL), Portugal (PT), Romania (RO), Slovenia (SI) and Slovakia (SK). The ESCB dataset is not publicly available though and this paper cannot disclose data by country. The sample period spans from 2007 to 2015, both years inclusive. This constitutes a panel dataset of 22 countries over 9 years. Policy actions are quantified as the additional annual public expenditure compared to previous year budget and expressed in millions of national currency. To have a consistent variable across Member States, I converted the policy actions to millions of euros of 2015 per capita.

**Fig. 1:**
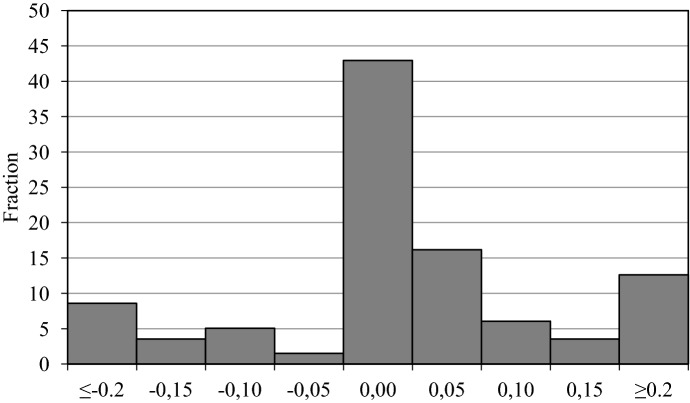
Histogram of all changes in old age pensions. Notes: measures as percentage of previous period nominal GDP. All countries, 2007–2015

Panel data offers regression analysis that the short time dimension of the dataset rules out by country. Although the sample period is admittedly short, the sample of countries and time period covered presents a rich amount of variation, essential for adequate regression analysis. Figure 1 shows the heterogeneity present in the sample with a histogram covering the entire sample of countries over the period 2007–2015. The measures range from less than − 0.2 to more than 0.2 of GDP. Around 43% of the observations are zero. There are also a significant number of nonzero observations; there are more pension increases than pension cuts.

The sign variation of the measures might reflect particularities of the sample period. During the first years we find a number of measures taken in response to the economic and financial crisis by the EU Member States. Since 2010, the EU Member States implemented austerity programmes to deal with inherited fiscal deficits and to improve the confidence in their economies to reduce borrowing costs. In some countries, long-run issues such as demographic trends or an ageing population were also addressed. Throughout the sample period, we also find increases with an ideological motivation or as means to improve the welfare insurance provided to vulnerable groups and individuals with l .

As explained earlier, a number of fiscal policy actions can be argued to be systematically related to the current state of the economy. In contrast, the identifying assumption to produce unbiased estimates of the aggregate effects of transfers shocks is that discretionary changes in old age pensions are exogenous. The ESCB dataset records discretionary changes in transfers relative to a ‘neutral policy’ benchmark. In other words, the compiled fiscal actions directly account for developments in GDP, inflation, or more generally, the level of economic activity. The next step is to identify the discretionary fiscal actions motivated by factors other than a systematic response to the current state of the economy.

### Narrative analysis

The ESCB dataset contains a description for all measures. The descriptions are a valuable source of information about the motivation behind the transfers changes. Whenever the descriptions were too short or imprecise, I complemented the available information with the narrative record. Among others, I consulted country-specific legislation and government reports, several papers and reports on behalf of the European Commission, and country reports by the IMF and the OECD. Occasionally, I also consulted news from sources such as The Wall Street Journal or the Economist. A full list of all complementary sources for the narrative analysis can be found in ‘Appendix’. The narrative analysis reclassifies the discretionary changes as either exogenous or not exogenous assigning them to one of the following categories:

- *Cyclical* This category includes changes in transfers due to current macroeconomic developments or within a package of opposing fiscal measures. For example, changes in transfers to promote short-run economic growth or to compensate for a tax hike or other public expenditures cuts. Deficit reduction actions are also classified as cyclical when they respond to short-run movements in the deficit or to offset another shock. Moreover, my classification follows a conservative approach that may over-classify the fiscal actions as countercyclical. While reducing the accuracy of the point estimates, this is done on the basis of obtaining unbiased estimates.
- *Reform* The most clearly exogenous reforms are policy actions to deal with demographic trends, or an ageing population. Following Cloyne (2013), this category also includes deficit consolidation actions to guarantee the long-run sustainability of public finances that were taken independent of the current macroeconomic situation. ‘Reforms’ also include policy actions imposed on policymakers by external bodies such as European rules or court rulings. I also include reforms for efficiency gains such as combining different transfers into a unique benefit, or to avoid incorrect receipt of benefits from those who actually do not meet the eligibility criteria when they are not a clear consequence of current macroeconomic developments.
- *Purchasing Power* policy actions to maintain and improve the purchasing power and living standards of beneficiaries. Includes those changes that, according to the established rule for adjustments, change transfers above or below the price index of reference. Also includes discretionary changes in transfers, usually targeted to low-income individuals, which increase the insurance provided by the welfare system. In other words, changes in transfers with an ideological motivation of fairness.^3^

In total I identify 177 policy changes. I find 44 ‘endogenous’ changes and 133 ‘exogenous’. Within the later, 59 changes were motivated by purchasing power reasons and 74 were the result of a reform.

**Table 1:** Predictability tests

	Output	Inflation	Unemployment rate	ALTR	Primary surplus
All changes	0.28	0.68	0.23	0.36	0.47
Exogenous	0.38	0.88	0.19	0.54	0.56

*p* values for Granger causality tests. A shorthand for the aggregate variable is stated at the top. A shorthand for the transfer shock is stated on the left. Regressions include one lag of the transfers shock and the selected aggregate. All regressions include country and year fixed effects. Estimation is by least squares and standard errors are clustered by country. Sample 2007–2015

### Predictability tests

If exogenous changes were in fact the response to other influences on output growth, it is likely that these discretionary changes could be predictable by proxies for those influences. This section tests this possibility following the standard practice in the narrative literature (Romer and Romer 2010; Mertens and Ravn 2012; Gil et al. 2019).

To test whether changes in transfers are predictable, I regress the discretionary changes on their own lag and a lag of output, inflation, the unemployment rate, the implicit Average Labour Tax Rate (ALTR), or the primary surplus. The selected macroeconomic variables aim to capture short-run macroeconomic conditions in each EU State Member. The regressions include country and year fixed effects. Then, I compute the F-test under the null hypothesis that the macroeconomic variables do not Granger cause the discretionary changes in transfers.^4^ A high significance level implies that we cannot reject the null. Table 1 shows the p-value for each test. The exogenous changes in old age pensions cannot be predicted by the selected indicators. Moreover, excluding ‘endogenous’ changes improves the tests results for several macroeconomic variables.

## Econometric framework

This paper estimates the aggregate effects of government income transfers shocks using policy actions for old age pensions. In the context of dynamic linear panel regression models consider the following baseline specification:

1 $$\begin{aligned} \text {ln} y_{it} = \alpha _i + \delta _t +\rho \text {ln} y_{it-1}+ \beta \Delta T_{it} + \gamma X_{it} + \varepsilon _{it} \end{aligned}$$

where the macroeconomic variable of interest $y_{it}$ for country *i* and year *t* is expressed in logs. The specification includes a lag of the dependent variable to capture dynamics in the relationship between transfers and the macroeconomic variables.^5^$\Delta T_{it}$ refers to the new narrative series of government income transfers shocks. $100 \cdot \beta$ measures the average percentage increase in a macroeconomic variable of interest caused by a unit increase in old age pensions. $\alpha _i$ represents the unobserved heterogeneity, $\delta _t$ year fixed effects. $X_{it}$ includes a set of control variables to be discussed below. Finally, $\varepsilon _{it}$ stands for the idiosyncratic error term.

The strategy to deal with the potential endogeneity of $\Delta T_{it}$ consists of applying a narrative analysis to the measures compiled in the ESCB dataset. The new measure of government income transfer shocks is most likely to satisfy the identifying assumption that transfers shocks are exogenous. First, the ESCB dataset compiles discretionary changes relative to a ‘neutral policy’ benchmark. That is, the measures directly account for short-run macroeconomic developments. Second, and most important, the narrative analysis excludes from these discretionary changes those systematically correlated with the current state of the economy.

Specification (1) also includes controls for other influences that might affect both, the outcome variables and transfers changes but may not be explicitly explained in the narrative record. Alternatively, we can think of the inclusion of control variables as a refinement to guarantee unbiased estimates. First, I include government spending and the implicit ALTR (inclusive of social security contributions) to control for spending in other public expenditures and how discretionary changes in transfers are financed. ‘Appendix’ presents the results from regressions that use alternative variables to control how discretionary changes in transfers are financed.^6^ Second, several changes in old age pensions correspond to inflation adjustments. Discretionary changes in transfers are measured in deviation to the standard evolution of prices in each country, but accidental correlation with other factors that affect both, the outcome variables and the changes in pensions due to inflation is always a possibility. Then, it is important to include the lag of the price level in the regressions.^7^ Moreover, the set of controls also includes a proxy for the monetary policy stance. The majority of countries belong to the Euro-area and have their interest rate of reference set by the European Central Bank. However, Slovakia is a Euro area member since 2009, while Bulgaria, the Czech Republic, Hungary, Poland and Romania have their interest rate of reference set by their respective National Central Bank.^8^ Finally, under the assumption that changes in international confidence are a common shock to all countries, they are captured in the year fixed effects. Any country-specific fixed deviations from the international sentiment would be captured in the country fixed effects.

The macroeconomic variables of interest are output, non-durables goods consumption, services consumption, durable goods consumption and private investment. All variables are in real and per capita terms.^9^ I also investigate the effects of transfers shocks to selected labour market indicators, which include employment per capita, hours per worker, the unemployment rate and the real wage.^10^ The measures of transfers shocks are available at annual frequency from 2007 to 2015. The rest of variables are available from 2005.

## The aggregate effect of transfers shocks

**Fig. 2:**
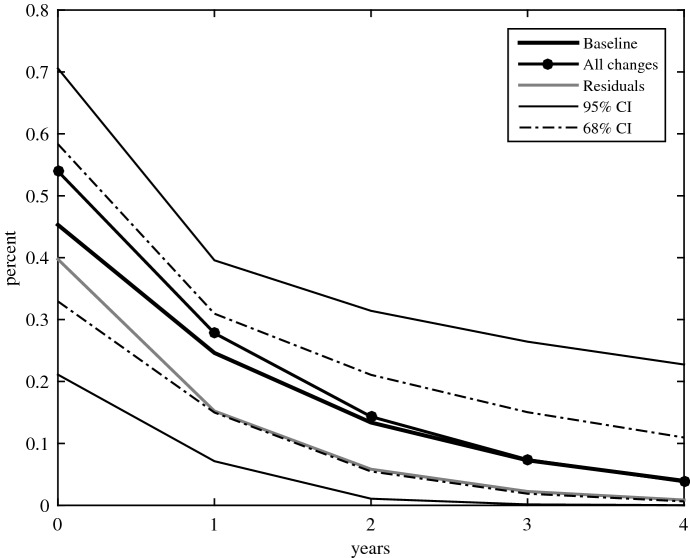
Dynamic response of output to transfers shocks. Notes: response to an increase in old age pensions equivalent to 1% of GDP. Transfer shocks are the narrative variable including only exogenous changes (black), all changes (marker), or residualised (grey). Full lines are point estimates; thin and broken lines indicate one and two standard deviations confidence intervals, respectively

I start estimating specification (1) for output as the outcome variable. Figure 2 shows the response of output to an increase in old age pensions. Multiplier effects are obtained with a shock to old age pensions equivalent to the value of 1% of median GDP in the sample and normalised by the ratio of GDP-to-old age pensions. The plot also reports bootstrap computed confidence intervals at the 95 and 68% confidence level.^11^ Transfers shocks in the baseline specification are the narrative variable including only exogenous changes in old age pensions (black lines).

The estimated multiplier effect for the baseline specification is between 0 and 1. On impact, output rises 0.45%. Thereafter, the effect of transfers shocks also includes the effect through lagged output. After one year, about half of the initial effect has faded and the multiplier takes the value of 0.25%. After three years the multiplier is statistically not different form zero. An alternative measure of the long-run effect of transfer shocks would be the long-run cumulative multiplier. This can be calculated as the sum of the impact responses of output until the effect of the shock dies out.^12^ The estimated long-run multiplier effect is close to 1.

In line with Párraga-Rodríguez (2018), using all discretionary changes overestimates the short-run effect of transfer shocks on output (circle marker). Output rises 0.54% upon impact. However, the multiplier is not statistically different from zero by the third year. The resultant long-run multiplier is slightly above unity and takes the value of 1.1%. The sign of the bias suggests a positive correlation between the estate of the economy and changes in old age pensions. Estimates that use all discretionary changes could be attributing to increases in transfers what actually would be the result of concealed factors associated with better financing capacity. The estimates do not differ significantly though. This could reflect the pre-treatment of policy actions in the ESCB dataset because policy actions are measured relative to a ‘neutral policy’ benchmark.

As a robustness check, I also present estimates for an alternative measure of the shocks based on the residuals of regressing all discretionary changes in transfers on a constant and a lag of output (grey line). That is, the alternative measure of transfer shocks removes predictable responses to output from the discretionary changes in transfers. The point estimates for this alternative measure are below the baseline estimates the entire forecast horizon. Output increases 0.40% upon impact, and the long-run multiplier effect is 0.6%. However, the differences are not statistically significant either.

At this point it is imperative in comparison with other estimates of the multiplier effect in the existing literature (although these measures do not afford a one-to-one comparison in all cases). In related research I estimate the dynamic aggregate effects of innovations to social security benefits in the USA during the period 1951–2007 (Párraga-Rodríguez 2018). Then I found an impact multiplier of 0.2, rising to an accumulated response of 1.0 after four quarters and a maximum value of 2.2 in the long run. With the same methodology, Gechert et al. (2016) estimate a multiplier effect of shock to social security benefits between 0 and 1 in Germany. They point out that the different estimates for US and European data could be due to a higher ratio of imports-to-GDP in Europe compared to the USA. In Párraga-Rodríguez (2022) I also found a multiplier effect of shock to social security benefits between 0 and 1 in Spain. Other comparable estimates are those for the tax multiplier. The following estimates are based on US data. In the SVAR tradition and for total tax revenues, Blanchard and Perotti (2002) find a peak multiplier of 0.8. Using sign restrictions in an SVAR framework, Mountford and Uhlig (2009) also estimate the effect of aggregate taxes and find an impact multiplier of 0.3, which rises to 0.9 after one year and reaches a maximum value of 3.4 after twelve quarters. Romer and Romer (2010) construct a narrative variable of legislated tax changes in the USA and estimate that a tax hike of 1% of GDP has a small and not statistically significant effect on output on impact, but maximum effect of 3.1% after ten quarters. Mertens and Ravn (2013) estimate the proxy SVAR for personal income taxes and find a multiplier of 2.0 on impact, rising to a maximum of 2.5 in the third quarter. Finally, Ramey (2011) literature survey sets the range of estimates for the government spending multiplier from 0.6 to 1.8.

### Aggregate expenditure components

Government income transfers affect the macroeconomy through changing the disposable income of households and their spending decisions. Therefore, it is important to study the effect of transfers shocks to different expenditure components to better understand the point estimates for the output multiplier. To this end, the next outcome variables are aggregate private consumption of non-durables, services and durables, and aggregate private investment.

**Fig. 3:**
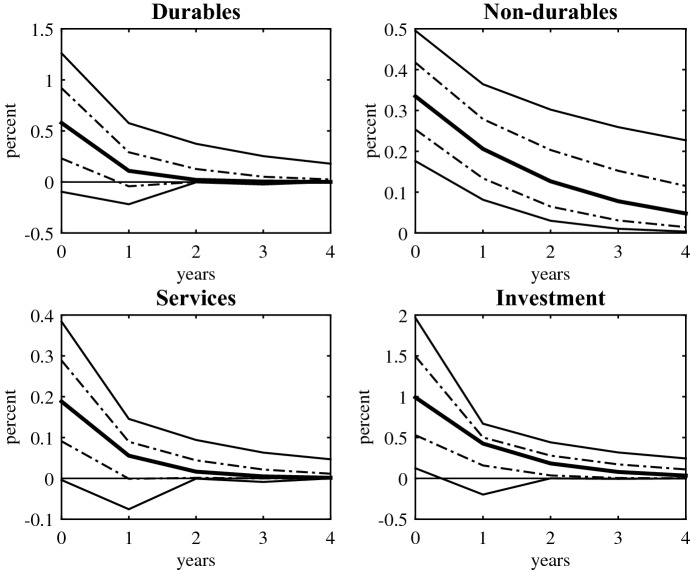
Dynamic response of aggregate expenditure components to transfers shocks

Figure 3 shows the dynamic response of aggregate expenditure components to an exogenous increase in old age pensions. The shocks are scaled to be equivalent to 1% of GDP. The plots also report 95 and 68% CIs. An increase in old age pensions yields a positive effect on all three aggregate consumption components. The larger response of durable goods consumption, 0.58%, than non-durables, 0.33%, or services, 0.19%, is in line with the existing literature. Evidence at the household-level predicts a larger response of durables than non-durables purchases to increases in disposable income.^13^ Moreover, Romer and Romer (2016) and Párraga-Rodríguez (2018, 2022) find that innovations to social security benefits trigger a larger response of durables purchases than non-durables consumption. However, the estimates for durables and services consumption are only significant at the 68% confidence level and transfer shocks have a longer lasting effect on non-durables consumption.

Finally, private investment rises 0.99% upon impact. Standard theory of the effect of public expenditure shocks predicts crowding out effects. However, unlike government spending, transfers do not compete directly with private spending. Government income transfers indirectly affect aggregate demand through redistribution. Moreover, this strong response of investment is in line with other estimates of the response of investment to tax shocks (Romer and Romer 2010). The estimates though are also imprecisely estimated; confidence intervals are wide on impact and, thereafter, the point estimates are not significant at the 95% confidence level.

**Fig. 4:**
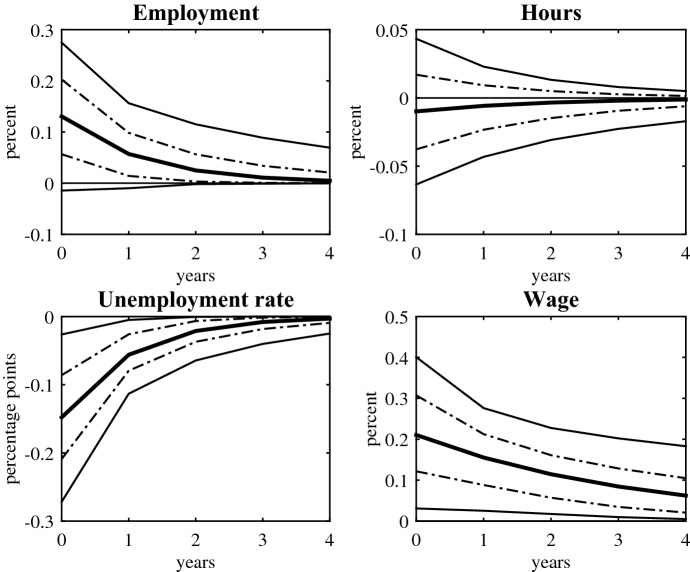
Dynamic response of labour market indicators to transfers shocks. Notes: response to an exogenous shock to old age pensions equivalent to 1% of GDP. Full lines are point estimates; thin and broken lines indicate 68 and 95% CIs, respectively

### Labour market indicators

Evidence on the aggregate effects of public expenditure shocks on the labour market is scarce: The limited existing literature has focused on the effects of government spending shocks.^14^ As an exception, Romer and Romer (2016) estimate with US data the effect of permanent increases in social security benefits on employment. This section extends the outcome variables to include hours per worker, the unemployment rate, and the real wage. The labour market indicators represent the extensive, intensive margins of labour, and a measure of labour costs.

Figure 4 shows the dynamic response of the selected labour market indicators to an increase in old age pensions. The shocks are scaled and equivalent to 1% of GDP. The plots also report 95 and 68% CIs. An increase in old age pensions has a positive effect on employment and the unemployment rate. This is consistent with the point estimates for the output multiplier and aggregate expenditure components. On the other hand, the response of hours is virtually zero and not significant. The estimates also indicate that increases in transfers are wage inflationary. The real wage rises 0.21% upon impact and the response is quite persistent. Overall though, and like Romer and Romer (2016), the size of the estimates is modest or imprecisely estimated.

## Estimates by motivation and regions

### Different motivations

The narrative analysis set three main motivations for transfers changes: cyclical conditions, reforms and the political will to sustain and improve the living standards of beneficiaries. Transfers changes in the last two categories are considered exogenous. Reforms include policies to guarantee the long-run sustainability of public finances, for efficiency gains or as a result of an external imposition on policymakers. ‘Purchasing power’ measures include those changes that, according to the established rule for adjustments, change transfers above or below the price index of reference. This category also includes changes with an ideological motivation of fairness or equity. However, changes associated with structural reforms usually involve transfers cuts while changes to improve the purchasing power of the beneficiaries usually involve increases. As a result, we might expect different effects from discretionary changes by motivation. This section investigates whether this is the case.

**Table 2:** The aggregate effect of transfers shocks by motivation

	Output	Consumption	Investment	Employment
Purchasing power	0.55	1.01	2.13	0.38
	(0.56)	(0.40)	(0.93)	(0.22)
Reform	0.46	0.21	0.89	0.11
	(0.14)	(0.08)	(0.48)	(0.06)
All exogenous	0.45	0.29	0.99	0.13
	(0.13)	(0.11)	(0.49)	(0.07)

A shorthand for the dependent variable is stated at the top of each column. A shorthand for the transfers shocks is stated on the left. The covariates include the lagged dependent variable, instrumented with the second lag. All regressions include country and year fixed effects, also include controls for monetary and tax policy. Estimation is by two-stage least squares and standard errors are clustered by country. The sample period is 2007–2015

Table 2 presents the results. To help in the comparison, I reproduce again estimates for the narrative variable which includes exogenous changes due to both motivations. The selected dependent variables summarise the aggregate effect of transfers shocks and include output, total private consumption expenditures, private investment, and employment per capita.^15^ Again, the coefficients correspond to the effect of an increase in old age pensions equivalent to 1% of GDP. Robust standard errors are in brackets and clustered by country. Comparing the second and third row in Table 2, the baseline point estimates are closer to estimates which only include ‘reform’ changes. This indicates that the baseline estimates might be mainly driven by changes due to reforms. On the other hand, estimates for ‘purchasing power’ changes have large standard errors. This imprecision could be partly attributed to the lower number of observations in this category. Nevertheless, once we account for the larger standard errors for the ‘purchasing power’ category, the point estimates for either motivation indicate similar positive aggregate effects.

### Different regions

This section relaxes the assumption of a single slope coefficient in specification (1) and presents estimates for the output multiplier in different regions. Pooled estimates measure the average effect of transfer shocks in EU Member States. However, the sample of countries presents differences like the degree of openness, the share of social expenditures or the number of retirees per capita that might affect the multiplier of transfers shocks. I establish three regions in line with EuroVoc’s definition of sub-regions in Europe. A Northern or continental region for AT, BE, DE, FR, FI, LU, NL. A Southern or Mediterranean region formed by CY, ES, GR, IT, PT, SI. The remaining countries form an Eastern European region: BG, CZ, HU, LV, PL, RO, SI, SK.^16^

**Table 3:** Multiplier effect by region

	Baseline	South	North	East
Impact effect	0.45	0.25	0.00	0.43
	(0.13)	(0.03)	(0.30)	(0.79)
Long-run effect	1.0	0.8	0.0	1.0

A shorthand for the region is stated at the top of each column. The covariates include the lagged dependent variable, instrumented with the second lag. All regressions include country and year fixed effects, also include controls for monetary and tax policy. Estimation is by two-stage least squares and standard errors are clustered by country. The sample period is 2007–2015

Table 3 compares the multiplier effects across regions caused by an identical increase in old age pensions in all regions. The shock to transfers is scaled to be equivalent to the value of 1% of median GDP and normalised by the ratio of GDP-to-old age pensions. For convenience, I reproduce again the baseline estimates for the pooled sample. The multiplier effect is the strongest in East Europe, while it is virtually zero in North Europe. The point estimates for these regions though have large standard errors and should be interpreted with caution. On the other hand, the output response is statistically significant for South Europe. An increase in old age pensions triggers a lower impact effect in South Europe compared to the baseline; however, the shock is more persistent and the resultant long-run multiplier effect of 0.8 is similar to the baseline estimates.^17^

## Conclusions

This paper has provided evidence on the aggregate effects of government income transfer shocks using a panel dataset of 22 EU Member States during 2007–2015. A contribution of this paper is the construction of a new measure of transfers shocks based on a dataset by public finance experts of the ESCB. The ESCB dataset records discretionary changes in old age pensions relative to a ‘neutral policy’ benchmark. A narrative analysis reclassifies these discretionary changes as either exogenous or not exogenous, i.e. a systematic response to the current state of the economy, according to their motivation.

A principal contribution of this paper is an estimate for the output transfers multiplier. The estimated old age pensions output multiplier ranges between 0 and 1. I also find a positive and significant effect of transfers shocks to aggregate expenditure components. On the other hand, the estimates indicate a positive though modest effect on the labour market. Estimates were also broken down by main motivation behind the policy actions and for three geographic regions, i.e. North, South and East Europe.

Finally, these results have important policy implications. A multiplier effect between 0 and 1 indicates limited effectiveness of fiscal actions involving transfers. However, this limited effectiveness might not have the same implications for stimulus and austerity programmes. On the one hand, the results indicate that increases in old age pensions might be costly stimulus measures given their modest positive impact. On the other hand, desirable austerity programmes should include measures that effectively reduce the government deficit while having a contained negative effect on the real economy. To draw stronger conclusions a larger panel either in terms of time span and/or number of countries seems the most promising way.

## Figures and Tables

**Fig. 5:**
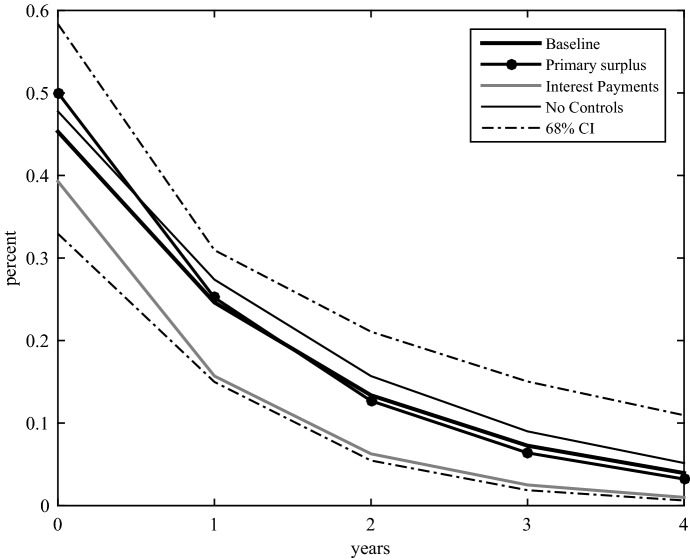
Notes: response to an exogenous shock in old age pensions equivalent to 1% of GDP. Full lines are point estimates; broken lines indicate 68% CI for baseline estimates

## Data Availability

The data supporting the findings of this study are available from the Working Group of Public Finance of the European System of Central Banks. However, restrictions apply to the availability of these data, which were used under license for the current study, and so are not publicly available. Data are, however, available from the authors upon reasonable request and with permission of the ESCB-WGPF.

## Appendix

### A1 References for the narrative analysis

Barr, N., and Diamond, P. (2015). ‘Italy’s pension reforms: facing the facts’ . Italy24, 15.05.2015.

Blommesteijn, M. (2013). ‘Assessment of the implementation of the European Commission. Recommendation on Active Inclusion. A Study of National Policies. The Netherlands’. In behalf of The European Commission.

Bloomberg Business. (2014). ‘Greece Meets Budget Target for More Debt Relief, EU Says’. News 23.04.2014.

BOE (2009). ‘Royal Decree-Law 10/2009, of 13th August, whereby the temporary unemployment protection and insertion program is regulated’. BOE-A-2009-13496.

BOE (2010).‘Royal Decree-law 8/2010, of 20th May, whereby extraordinary measures to reduce the public deficit are adopted’. BOE-A-2010-8228.

BOE (2011).‘Royal Decree-law 20/2011, of 30th December, whereby urgent measures on public finances are adopted to reduce the public deficit’. BOE-A-2011-20638.

BOE (2010).‘Royal Decree-law 20/2012, of 13th July, about measures that guarantee the budget stability and promote competition’. BOE-A-2012-9364.

Czech Republic (2008) National Reform Program of the Czech Republic 2008–2010.

Czech Republic Ministry of Labour and Social Affairs (2014) ‘Basic indicators of labour and social protection in the Czech Republic. Time series and graphs 2013’.

Dvorakova, Z. and Stroleny, A. (2012) Social dialogue and the public services in the aftermath of the economic crisis: strengthening partnership in an era of austerity in the Czech Republic. National report. European Commission project. VP/2011/00.

De Nederlandsche Bank, Annual Report 2007 and 2008.

Embassy of France in London (2012) ‘The French pension reform-key elements’. France in the UK news, May 2012.

European Commission. (2010) ‘The economic adjustment programme for Greece’. Directorate-General for Economic and Financial Affairs. Occasional Papers 61.

European Commission. (2013). ‘Old-age benefits: Commission refers Slovakia to Court for refusing to pay an old-age benefit to pensioners abroad’. Press Release, Brussels, 25.04.2013.

European Economy. (2011) ‘The economic adjustment programme for Portugal’. Directorate-General for Economic and Financial Affairs. Occasional Papers 79.

European Economy. (2014) ‘The economic adjustment programme for Portugal 2011–2014’. Directorate-General for Economic and Financial Affairs. Occasional Papers 202.

Fornero, E. (2013). ‘Italy’s Reforms Are Bearing Fruits’. Published in the Wall Street Journal, Opinion Europe. 05.06.2013.

Hartz concept: https://en.wikipedia.org/wiki/Hartz_concept

Hellenic Republic Ministry of Finance. (2011). Medium Term Fiscal Strategy 2012–2015.

Hellenic Republic. (2011). Letter of Intent, Memorandum of Economic and Financial Policies, and Technical Memorandum of Understanding.

Hungary Central Administration of National Pension Insurance. (2013) ‘Eligibility criterion/retirement age, service time and calculation of the pension amount’. 08.03.2013. www.onyf.hu

International Monetary Fund (2010) ‘Bulgaria: Selected Issues’. IMF Country Report No. 10/159.

International Monetary Fund (2009) ‘Austria: 2009 Article IV Consultation’. IMF Country Report No. 09/295.

International Monetary Fund (2010) ‘Bulgaria: Selected Issues’. IMF Country Report No. 10/159.

International Monetary Fund (2010) ‘Bulgaria: Reforming the pensions system’. November 2010.

International Monetary Fund (2015) ‘Bulgaria: 2015 Article IV Consultation’. IMF Country Report No. 15/119.

International Monetary Fund (2014) ‘Cyprus: Selected Issues’. IMF Country Report No. 14/314.

International Monetary Fund (2007) ‘Cyprus: Selected Issues’. IMF Country Report No. 07/71.

International Monetary Fund (2010) ‘Czech Republic: Staff Report for the 2010 Article IV Consultation’. IMF Country Report No. 10/60.

International Monetary Fund (2014) ‘Czech Republic: 2014 Article IV Consultation’. IMF Country Report No. 14/256.

International Monetary Fund (2013) ‘Czech Republic: 2013 Article IV Consultation’. IMF Country Report No. 13/242.

International Monetary Fund (2014) ‘Czech Republic: 2014 Article IV Consultation’. IMF Country Report No. 14/256.

International Monetary Fund (2014) ‘France: 2014 Article IV Consultation’. IMF Country Report No. 14/182.

International Monetary Fund (2009) ‘Greece: 2009 Article IV Consultation’. IMF Country Report No. 09/244.

International Monetary Fund (2006) ‘Hungary: 2006 Article IV Consultation’. IMF Country Report No. 06/379.

International Monetary Fund (2013) ‘Hungary: 2013 Article IV Consultation’. IMF Country Report No. 13/85.

International Monetary Fund (2012) ‘Republic of Latvia: Request for Stand-By Arrangement-Staff Report; Staff Supplement; Press Release on the Executive Board Discussion; and Statement by the Executive Director for the Republic of Latvia’. IMF Country Report No. 09/3.

International Monetary Fund (2012) ‘Romania: 2012 Article IV Consultation and Sixth Review Under the Stand-By Arrangement, and Requests for Waiver of Nonobservance of Performance Criterion and Modification of Performance Criteria’. IMF Country Report No. 12/290.

International Monetary Fund (2015) ‘Romania: 2015 Article IV Consultation’. IMF Country Report No. 15/79.

Ireland, Government of. (2011). Medium-Term Fiscal Statement. Government publications sale office, Prn. A11/1953.

Ireland, Government of. (2014) Budget 2014. Minister for Finance speech.

Ireland, Government of. (2009) Statement of the Minister for Finance. 7th April 2009.

Ireland, Government of. (2008) Statement of the Minister for Finance. 14th October 2008.

Investment & pensions Europe. (2010). ‘Portugal Telecom agrees pension transfer to state treasury’. 3 December 2010.

Kingdom of Spain (2015) Stability Programme Update 2015-2018.

Kingdom of Spain (2012) Stability Programme Update 2013-2016.

Kingdom of Spain (2007) Economic and Financial report on the government budget for 2007.

Kingdom of Spain (2012) Economic and Financial report on the government budget for 2012.

Kozek, W., Zieleńska, M. and Kubisa, J. (2013). ‘National report: Poland’. FP7 project ‘Combating Poverty in Europe: Re-organising Active Inclusion through Participatory and Integrated Modes of Multilevel Governance’.

Luxembourg, Government of. (2006). Government’s statement on the economic, social and fiscal 2006 countries (French translation).

Legros, M. (2009) Minimum Income Schemes. From crisis to another, The French experience of means-tested benefits. France. On behalf of European Commission, DG Employment, Social Affairs and Equal Opportunities.

National Bank of Belgium. (2007) ‘Report 2007. Economic and financial developments’.

National Bank of Belgium. (2008) ‘Report 2008. Economic and financial developments’.

Netherlands, Ministry of Economic Affairs. (2014).‘National Reform Programme 2014. The Netherlands’.

OECD. (2009). OECD Economic Surveys. Belgium. Volume 2009/12.

OECD. (2008). OECD Economic Surveys. Germany. Volume 2008/7.

OECD. (2010). OECD Economic Surveys. Germany. Volume 2010/9.

OECD. (2012). OECD Economic Surveys. Germany.

OECD. (2014). OECD Economic Surveys. Germany.

OECD. (2010). OECD Economic Surveys. Finland. Volume 2010/4.

OECD. (2016). OECD Economic Surveys. Finland.

OECD. (2007). OECD Economic Surveys. Greece. Volume 2007/5.

OECD. (2015). OECD Economic Surveys. Italy.

OECD. (2015). OECD Economic Surveys. Latvia.

OECD. (2008). OECD Economic Surveys. Luxembourg. Volume 2008/12.

OECD. (2010). OECD Economic Surveys. Netherlands. Volume 2010/10.

OECD. (2012). OECD Economic Surveys. Netherlands.

OECD. (2013). OECD Economic Surveys. Slovenia.

OECD. (2015). OECD Economic Surveys. Slovenia.

Schratzenstaller, M. (2009). ‘The Tax Reform 2009-10’. Austrian Economic Quarterly 4/2009.

Schratzenstaller, M. (2011). ‘Draft Federal Budget 2011’. Austrian Economic Quarterly 1/2011.

Slovenia, Government of the Republic of (2011). 152nd Government Session: Public Finance Act and Intervention measures for 2012.

The Economist (2014). ‘Italy’s budget. Mamma’s boy. Matteo Renzi gives mothers a tax break’. 25.10.2014.

Slovenia, Government of the Republic of . (2011). ‘152nd Government Session: Public Finance Act and Intervention measures for 2012’. Press Release, Republic of Slovenia, 29.09.11.

Wall Street Journal (2012) ‘Greece Passes 2013 Austerity Budget’. Europe. 12.11.2012.

Wall Street Journal (2011) ‘Belgium’s Di Rupo Sees Government Formed in Week’. New York, N.Y. 27.11.2011.

Werner Eichhorst, W., Gerard, M., Kendzia, M. J., Panayotova, N. and Vassileva, I. (2012) ‘Labour Market Situation and Pension System in Bulgaria’. European Parliament, Directorate General for Internal Policies, policy department a: economic and scientific policy, IP/A/EMPL/NT/2012-03.

Zasova, A., Krümina, M., and Rastrigina,O. (2012).‘The evolution of tax and benefit policy in Latvia: what has been the place of distributional considerations?’ Baltic Journal of Economics, 12(2), 5–16.

### A2 Policy actions in Spain

Next table summarises the source, motivation, and description for all Spanish policy actions reported in the fiscal questionnaires between 2007 and 2015. The implementation year and descriptions are directly taken from the fiscal questionnaires; all remaining information builds on the data reported in the questionnaires.

**Table:**

Year	Source	Policy action	Motivation	Notes and Methodology
2007	General State Budget Law 42/2006, 28 December	Improvement in minimum pensions, with an increase of 4.5% if the pensioner has a dependent spouse, 3% if he/she does not and 1 point for SOVI (compulsory old-age and disability insurance) pensions. These in addition to a 2% increase corresponding to the expected change in the CPI	Purchasing power	The benchmark for indexation of pensions is the rate of CPI inflation from November t-1 to November t. A measure is defined as the legislated pension increases above (below) this benchmark, multiplied by total expenditure in t-1. Minimum pensions have more often a final adjustment (including deviations of the expected change in the CPI from realised inflation) that deviate from the rate of CPI inflation
2008	General State Budget Law 51/2007, 26 December	Improvement in minimum pensions, with an increase of 6.5% if the pensioner has a dependent spouse, 5% if he/she does not, and 22.3% if the pensioner is a widower with family obligations. 1% increase of SOVI pensions above the expected change in the CPI	Purchasing power

**Table:**

Year	Source	Policy action	Motivation	Notes and methodology
2009	General State Budget Law 2/2008, 23 December	Improvement in minimum pensions, with an increase of 3% in addition to the increase due a deviation of the forecast from real inflation in the previous year. The rise is equivalent to additional 15 euros per month to minimum pensions for pensioners with dependent spouse, and pensioners that constitute a single economic unit of survivors, retirement or disability pensions. Guaranteed purchasing power to other pensioners with an increase of 2%, together with an increase due to a deviation of the forecast from real inflation in the previous year	Purchasing power	The benchmark for indexation of pensions is the rate of CPI inflation from November t-1 to November t. A measure is defined as the legislated pension increases above (below) this benchmark, multiplied by total expenditure in t-1. Minimum pensions have more often a final adjustment (including deviations of the expected change in the CPI from realised inflation) that deviate from the rate of CPI inflation
2010	General State Budget Law 26/2009, 23 December	Improvement in minimum pensions, with an increase of 2% on average for all minimum pensions from the Social Security and SOVI. The increase is equivalent to 15 euros per month for pensioners with dependent spouse or that constitute a single economic unit	Purchasing power

**Table:**

Year	Source	Policy action	Motivation	Notes and methodology
2010	Royal Decree-Law 8/2010, 20 May	Withdrawal of transitory regime for partial retirement	Reform	The benchmark is the hypothetical counterfactual of no change in the legislation. The measure is defined as the difference in expenditure from what this would have been absent the change in the legislation. Estimation from official source. Policy action due to an ‘external’ imposition: ‘[...] speed, security and determination in action is part of the commitment of the member countries of the euro zone to strengthen confidence in the single currency and the stability of the euro zone’
2011	Royal Decree-Law 8/2010, 20 May	Pensions freeze	Reform	Legislation implies measures to reduce the public deficit due to an ‘external’ imposition: ‘The measures outlined require the adoption of a legal rule. The need for immediate application in some cases, to ensure their effectiveness in reducing spending, and its realisation, knowledge and security in other, so that their credibility and immediate effect on financial transactions and the relevant actions to guarantee for the stability of the euro [...]’

**Table:**

Year	Source	Policy action	Motivation	Notes and methodology
2012	Royal Decree-Law 20/2011, 30 December	No adjustment in pensions for the deviation of forecast from real inflation in the previous year	Reform	Measures to reduce the public deficit due to an ‘external’ imposition: ‘Spain was granted an additional year, until 2014, to bring the deficit below 3% also modifying the deficit targets of the intervening years. This concession did not mean at all a relaxation but, on the contrary, a tightening of fiscal consolidation efforts’ (Stability Programme 2013–2016)
2013	General State Budget Law 17/2012, 27 December	Increase of pensions above CPI, with an increase of 1% for all pensions.	Cyclical	Percentage increase retrieved from the presentation project of the General State Budget. Increase in compensation for no adjustment due to a deviation of expected change in the CPI from real inflation in the previous year. In a press conference, 30 November 2012, vice-president Soraya Sáenz de Santamaría said: ‘We are well aware that you cannot ask all pensioners the same effort and that we must discuss the matter [no adjustment in pensions] with fairness, hence, in January 2013 pensions will be increased, in general, 1%, but 2% for pensioners who earn less than a thousand Euros’

### A3 Alternative controls for fiscal policy

Figure 5 presents estimates for multiplier effect and alternative controls for fiscal policy. The controls include the primary surplus (line with marker) and interest payments of outstanding debt (grey line). The primary surplus is defined as net lending/borrowing of general government (Eurostat’s series B9) minus interest payments (Eurostat’s series D41 PAY). To help in the comparison, black lines reproduce baseline estimates discussed in the main text. Figure 5 also includes estimates without controls for monetary or fiscal policy (thin black line) though all regressions include country and year fixed effects. The differences between coefficients are not statistically significant and range from 0.39 to 0.50 upon impact.

## Footnotes

1 Parker et al. (2006) study the effects of the 2001 tax rebates with similar findings.

2 Other transfers include benefits such as family/children, sickness, exclusion, disability, housing and health-care-related transfers.

3 Initially I broke down ‘Purchasing Power’ into changes motivated to keep up with inflation, and changes motivated by a political will to increase the safety net of the social security. There were too few observations per category to have meaningful variables.

4 Standard errors are clustered by country and are robust to heteroskedasticity and serial correlation.

5 Estimation is by two-stage least squares and instruments the lagged dependent variable with the second lag of $ln y_{it}$. I verified whether this is sufficient to avoid non-stationary values of $\rho$ close or larger than one. All estimates are well below unity, and Figs. 2, 3 and 4 show stationary dynamics. The inclusion of country fixed effects controls for unobserved heterogeneity.

6 The implicit ALTR is defined as total taxes on employed labour (Eurostat’s series D51A_C1, D29c and D611) divided by compensation of employees (Eurostat’s series D1) plus total wage bill and payroll taxes (Eurostat’s series D29c). Government spending stands for the sum of intermediate consumption, gross fixed capital formation and compensation of employees of the general government (data from Eurostat).

7 Series from Eurostat, All items HICP (2015=100).

8 Source: international financial statistics (IMF). Euro-area, Slovakia and Bulgaria interest rate of reference correspond to the Central Bank Policy Rate. The Czech Republic and Poland interest rate is the Repurchase Agreement Rate. The interest rate of reference for Hungary and Romania corresponds to the Discount Rate.

9 Output corresponds to gross domestic product at market prices (Eurostat’s national accounts). Consumption aggregates are retrieved from Eurostat’s final consumption aggregates by durability at market prices of non-durable goods, durable goods and services. Private investment corresponds to gross fixed capital formation at current prices of the private sector (AMECO series UIGP). Nominal variables are deflated with the HICP base 2015. Variables are converted in per capita terms dividing by total population (Ameco series NPTD). Data last retrieved in April 2016.

10 Employment per capita corresponds to total Employment (Ameco series NETD) divided by population; Hours per worker correspond to average annual hours worked per person employed (Ameco series NLHA) divided by 52; the unemployment rate corresponds to the Eurostat series une$\_$rt$\_$a; the real wage is nominal compensation per employee of the total economy (Ameco series HWCDW) divided by the HICP.

11 Robust standard errors to heteroskedasticity and serial correlation are clustered by country. The confidence interval for the impact responses are equivalent to adding or subtracting 1 or 2 standard deviations. Thereafter, confidence intervals are computed from 10,000 draws of $\beta$ and $\rho$ from a bivariate normal distribution with mean and covariance matrix equal to the point estimates and covariance matrix of the regression coefficients.

12 Formally, $m = \sum _{t=0}^{\infty } \rho ^t \beta = \frac{\beta }{1-\rho }$, where *m* denotes the long-run multiplier.

13 See, for example, Parker et al. (2006, 2013).

14 See Monacelli et al. (2010), Ravn and Simonelli (2007), Chodorow-Reich et al. (2012) and references therein.

15 Total aggregate consumption corresponds to the sum of non-durables, durables and services consumption.

16 IE, and MT are excluded due to lack of variation in discretionary changes for old age pensions.

17 As described earlier in the text, long-run effects are computed as the sum of output responses.

## References

[CR1] Blanchard O, Perotti R (2002). An empirical characterization of the dynamic effects of changes in government spending and taxes on output. Q J Econ.

[CR2] Bodkin R (1959). Windfall income and consumption. Am Econ Rev.

[CR3] Chodorow-Reich G, Feiveson L, Liscow Z, Woolston WG (2012). Does state fiscal relief during recessions increase employment? Evidence from the American Recovery and Reinvestment Act. Am Econ J Econ Pol.

[CR4] Cloyne J (2013). Discretionary tax changes and the macroeconomy: new narrative evidence from the United Kingdom. Am Econ Rev.

[CR5] Cuadro-Sáez L, López-Vicente FS, Rodríguez SP, Viani F (2020) Fiscal policy measures in response to the health crisis in the main euro area economies, the United States and the United Kingdom. Occasional paper 2019, Banco de España

[CR6] Gechert S, Paetz C, Villanueva P (2016) Top-down vs. bottom-up? Reconcilling the effects of tax and transfer shocks on output. IMK working paper 169-2016, Hans Boeckler Foundation, Macroeconomic Policy Institute

[CR7] Gil P, Martí F, Morris R, Pérez JJ, Ramos R (2019). The output effects of tax changes: narrative evidence from Spain. SERIEs J Span Econ Assoc.

[CR8] Hausman JK (2016). Fiscal policy and economic recovery: the case of the 1936 veterans’ bonus. Am Econ Rev.

[CR9] Jappelli T, Pistaferri L (2010). The consumption response to income changes. Ann Rev Econ.

[CR10] Kubota S, Onishi K, Toyama Y (2021). Consumption responses to COVID-19 payments: evidence from a natural experiment and bank account data. J Econ Behav Organ.

[CR11] Liu Q, Shen Q, Li Z, Chen S (2021). Stimulating consumption at low budget: evidence from a large-scale policy experiment amid the COVID-19 pandemic. Manag Sci.

[CR12] Mertens K, Ravn OM (2012). Empirical evidence on the aggregate effects of anticipated and unanticipated US tax policy shocks. Am Econ J Econ Pol.

[CR13] Mertens K, Ravn OM (2013). The dynamic effects of personal and corporate income tax changes in the United States. Am Econ Rev.

[CR14] Monacelli T, Perotti R, Trigari A (2010) Unemployment fiscal multipliers. Working paper 15931, National Bureau of Economic Research

[CR15] Morris R, Rizza P, Borgy V, Brandt K, Coutinho Pereira M, Jablecka A, Pérez JJ, Reiss L, Rasmussen M, Triki K, Wemens L (nd) Towards a (semi-)narrative analysis of fiscal policy in EU Member States. Technical report

[CR16] Mountford A, Uhlig H (2009). What are the effects of fiscal policy shocks?. J Appl Econom.

[CR17] Oh H, Reis R (2012). Targeted transfers and the fiscal response to the great recession. J Monet Econ.

[CR18] Parker JA, Johnson DS, Souleles NS (2006). Household expenditure and the income tax rebates of 2001. Am Econ Rev.

[CR19] Parker JA, Souleles NS, Johnson DS, McClelland R (2013). Consumer spending and the economic stimulus payments of 2008. Am Econ Rev.

[CR20] Párraga-Rodríguez S (2018). The dynamic effects of public expenditure shocks in the United States. J Macroecon.

[CR21] Párraga-Rodríguez S (2022). A raise for grandma: pensions and household expenditure. Econ J.

[CR22] Poterba J (1988). Are consumers forward looking? Evidence from fiscal experiments. Am Econ Rev.

[CR23] Ramey VA (2011). Can government purchases stimulate the economy?. J Econ Lit.

[CR24] Ramey VA (2019). Ten years after the financial crisis: what have we learned from the renaissance in fiscal research?. J Econ Perspect.

[CR25] Ravn MO, Simonelli S (2007) Labour market dynamics and the business cycle: Structural evidence for the United States. Discussion paper DP6409, CEPR

[CR26] Romer C, Romer D (2010). The macroeconomic effects of tax changes: estimates based on a new measure of fiscal shocks. Am Econ Rev.

[CR27] Romer C, Romer D (2016). Transfer payments and the macroeconomy: the effects of social security benefit increases, 1952–1991. Am Econ Rev.

[CR28] Stephens MJ (2003). 3rd of the month: do social security recipients smooth consumption between checks?. Am Econ Rev.

[CR29] Wilcox DW (1989). Consumption and liquidity constraints: an empirical investigation. J Polit Econ.

